# Cryptic pulmonary cryptococcosis: An atypical presentation of pulmonary cryptococcosis in a HIV-infected patient

**DOI:** 10.7196/AJTCCM.2019.v25i4.002

**Published:** 2019-12-06

**Authors:** H M Maepa

**Affiliations:** Division of Pulmonology, Chris Hani Baragwanath Academic Hospital, and School of Medicine, Faculty of Health Sciences, University of the Witwatersrand, Johannesburg, South Africa

**Keywords:** pulmonary crptococcosis, HIV, disseminated cryptococcus, atypical

## Abstract

Pulmonary cryptococcosis is a fungal infection caused by inhalation of *Cryptococcus gattii* and/or *C. neoformans* spores. It mostly affects HIV-infected patients. This is a case report of a severely immunocompromised HIV-infected patient, presenting with respiratory symptoms and atypical chest X-ray features for pulmonary cryptococcosis. A serum cryptococcal latex antigen test is positive in a majority of HIV-infected patients with pulmonary cryptococcosis. This case report demonstrates the occurrence of a false negative serum cryptococcal latex antigen test, becoming positive with the development of an unmasking immune reconstitution syndrome (after antiretroviral therapy was commenced). This also resulted in the characteristic cryptococcal lung cavities observed on computed tomography chest images. Duration of fluconazole therapy should be individualised, and serial chest imaging (e.g. chest X-ray) should be performed to monitor treatment response

## Background

Pulmonary cryptococcosis is a fungal infection mainly caused by *Cryptococcus gattii* and/or *C. neoformans*. The fungus causes pulmonary infection when its poorly encapsulated spores (basidiospores), found worldwide in soil contaminated by avian droppings, are deposited in the alveoli and terminal bronchioles of the lung after inhalation.^[1]^

Pulmonary cryptococcosis is less frequent when compared to cryptococcal meningitis (CCM).^[1,2]^ It is more common in patients with AIDS, with a more aggressive course and a high propensity to disseminate to the meninges.^[3]^ This indicates that HIV-infected patients with pulmonary cryptococcosis invariably also have coexisting extra-pulmonary cryptococcal disease such as CCM, and /or other systemic cryptococcal manifestations, e.g. bone marrow infiltration.^[1,2]^

## Case

Ms PM is a 28-year-old female, originally from Eastern Cape Province in South Africa (SA). She was first admitted in July 2017 for severe respiratory distress at a district hospital in Gauteng Province, with a working diagnosis of multi-lobar pneumonia. Further examination revealed significant wasting, pallor and oral candidiasis. Her oxygen saturation was 82% on room air, which improved to 100% on supplemental oxygen. She was hypotensive (blood pressure 89/65 mmHg), and tachycardic (120 beats per minute) due to sepsis.

**Fig. 1:**
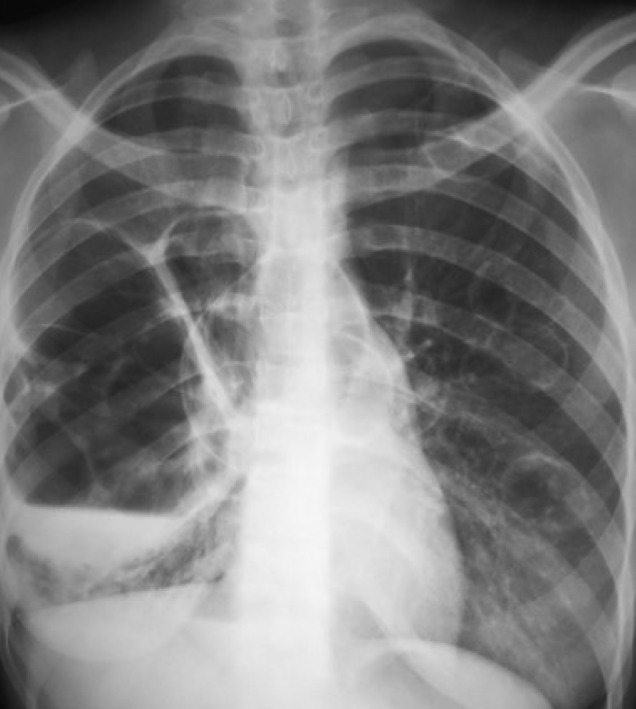
Chest X-ray showing multiple cysts.

Her chest X-ray showed diffuse, multiple thin-walled cysts (one with air-fluid level) of varying sizes in both lung fields; as well as the peri-cardiac region (Fig. 1). Due to the patient’s rural background, hydatid lung disease was now the working diagnosis. A computed tomography (CT) scan of the chest could not be performed at this district hospital as the scan machine was not operational.

Blood results showed acute kidney injury (resolved with crystalloid fluids), a raised c-reactive protein (CRP) of 143 mmol/L, and a normocytic anaemia attributed to her HIV infection. The hydatid serology and the serum cryptococcal latex antigen test (sCLAT) performed was negative. Sputum investigations were negative for tuberculosis, yeast and/or fungal species. Bronchoscopy equipment was not available at this hospital, and no further biological samples could be collected.

## Treatment and clinical course

Therapeutic oral albendazole therapy was started for potential hydatid lung disease, oral fluconazole therapy for severe oral candidiasis, and intravenous augmentin for superimposed bacterial infection. Albendazole therapy was continued despite negative results owing to the suspected low sensitivity of hydatid serology tests currently available in SA. Considering the patients’ immunocompromised state, it was important to treat the potential parasitic infection.

The patient showed marked clinical improvement during her hospitalisation, and was discharged after 10 days. Antiretroviral therapy (ART) was initiated prior to discharge. She was discharged on ART, 200 mg fluconazole orally daily (for oral candidiasis), and 800 mg albendazole orally daily (for 28 days) for suspected hydatid lung disease. She was to follow up in one month at the respiratory outpatient department at Chris Hani Baragwanath Academic Hospital (CHBAH), where her contrast CT scan of the chest was to be performed and reviewed.

At her 1-month follow-up at CHBAH (patient had been on ART for 1 month) she was clinically well, with no residual respiratory symptoms. However, she complained of severe intractable headache suggestive of meningitis. A repeat sCLAT as well as a lumbar puncture was performed. Lumbar puncture confirmed CCM, with a cerebrospinal fluid CLAT titre of 2 048. The repeat sCLAT was now positive with a titre of 2 000. Ms PM was immediately readmitted with a working diagnosis of unmasking immune reconstitution infIammatory syndrome (IRIS) in the form of disseminated cryptococcal infection (pulmonary cryptococcosis as well as CCM).

**Fig. 2:**
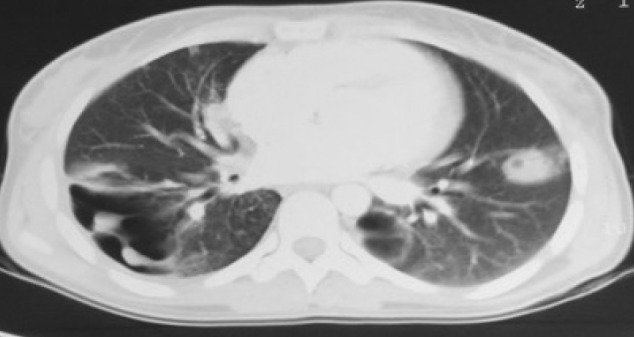
Computed tomography chest scan showing multiple thick-walled cavities.

Review of the CT scan of the chest supported the diagnosis of pulmonary cryptococcosis, which now revealed multiple, bilateral thick-walled cavities (not cysts as initially observed on the chest X-ray) in both lung fields (Fig. 2). This radiological change was not in keeping with hydatid lung disease, and therefore albendazole therapy was discontinued. Intravenous amphotericin B therapy was commenced, and fluconazole therapy was continued at meningitis doses of 400 mg via intravenous injection (IVI) 12-hourly.

### Outcomes

**Fig. 3:**
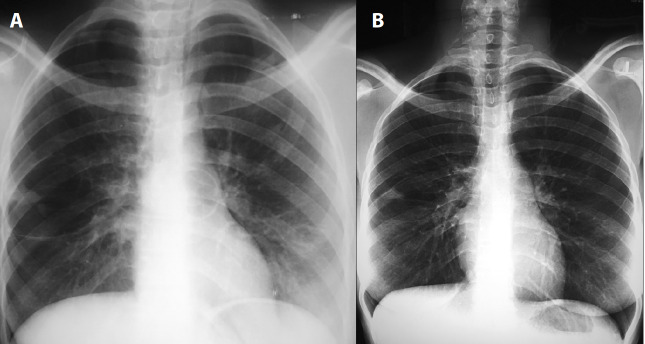
(A) Resolving lung opacities 3 months after starting fluconazole therapy. (B) Normal chest X-ray 6 months after starting treatment.

**Fig. 4:**
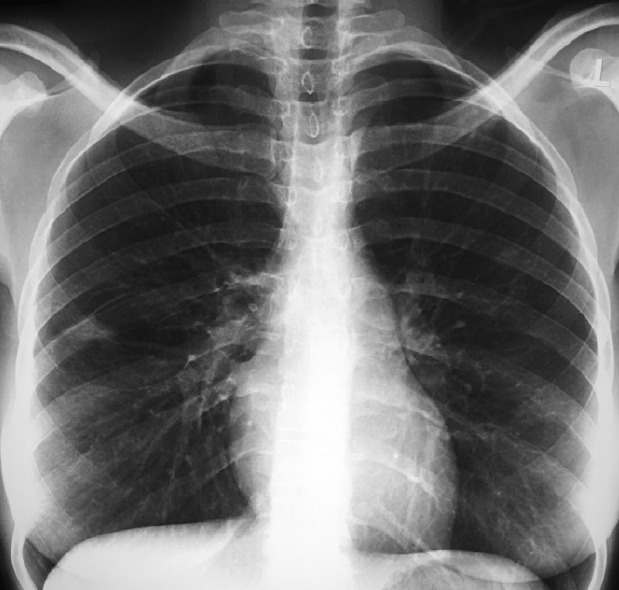
Chest X-ray at completion of treatment.

Ms PM had an uneventful second hospital admission and was discharged with a CD4 count of 48 cells/μL. She continues to do well. Her follow-up chest X-ray at 3 months showed a significant reduction in the number of lung opacities observed (Fig. 3). She continued with fluconazole therapy at 400 mg per os daily for a further 4 months (total of 6 months) with total resolution of lung opacities by the end of 6 months (Fig. 4). She continues to take 200 mg fluconazole orally daily until her CD4 lymphocyte count is greater than 200 cells/μL.

## Discussion

An sCLAT is positive in a majority of patients with HIV infection and pulmonary cryptococcosis, and should prompt physicians to investigate for disseminated disease. It is therefore an excellent screening test in immunocompromised patients with respiratory symptoms.^[1,2]^ Our patient initially displayed a false-negative sCLAT with chest X-ray images not typical for the diagnosis of pulmonary cryptococcosis. However, the development of unmasking IRIS post initiation of ART in our patient (a month after she was discharged from hospital) unveiled the true severity of disseminated cryptococcal disease which was initially difficult to diagnose. The extent and severity of disseminated cryptococcal disease is best correlated with CD4 counts less than 100 cells/μL and sCLAT titres higher than 1:256. ^[1,2,4,5]^ Ms PM had a CD4 count below 50 cells/μL, and her repeat sCLAT was positive with titres above 2 000. These results appropriately correlated with severity and dissemination of her infection, requiring immediate treatment.

The false-negative sCLAT result (initially seen in our patient) can occur in samples with a large amount of antigen, as it is processed with latex agglutination.^[1]^ False-positive results also occur in patients infected with other fungal and/or bacterial organisms such as *Trichosporon asahii* and *Stomatococcus*. ^[1]^ Visualisation of encapsulated yeast formed in sputum, broncho-alveolar lavage or tissue specimens via bronchoscopy is also diagnostic of cryptococcal pulmonary infection.^[1,6]^ Sputum cultures performed for Ms PM were negative for yeasts and/or fungal species. Bronchoscopy apparatus, as well as laboratory personnel accustomed in evaluating bronchoscopy tissue or lavage specimens, are mostly unavailable in many district hospitals in Gauteng. This poses another challenge in rapid and correct diagnosis of these patients.

The most common radiological feature of pulmonary cryptococcosis is clustered nodules, with an upper and midzone lung distribution bilaterally. Cavitation may may be seen in up to 40% of cases. Scattered nodules, a solitary pulmonary nodule or mass with or without cavitation, and peribronchovascular consolidation are less common.^[2,5,6]^ The initial chest X-ray (Fig. 1) of our patient did not have the common nodules or cavitations expected with pulmonary cryptococcosis. It was unfortunate that a contrast CT scan of her chest could not be performed while being admitted, as the scan machine at the admitting hospital had broken down. This is a major challenge in resource-limited facilities where such important equipment is not regularly maintained. The eventual cavities seen on the CT scan of her chest (Fig. 2) were diagnostic.

The optimal treatment of pulmonary cryptococcosis is unknown. It is mainly extrapolated from our management of CCM. The main goal of treatment is to control signs and symptoms of pulmonary cryptococcosis and reduce dissemination to the central nervous system. Treatment must immediately be initiated in patients with severe pulmonary disease (e.g. diffuse pulmonary infiltrates, cavities) or disseminated disease with a sCLAT titre ≥1:512 to avoid poor outcomes.^[1,7]^ Treatment consists of 400 mg fluconazole (6 mg/ kg orally daily) for 6 to 12 months. Intravenous amphotericin B should be added to therapy when CCM is also diagnosed, as was done with our patient.^[1,7]^ Alternative agents used in the treatment of pulmonary cryptococcosis include itraconazole, voriconazole, posaconazole and isavuconazole which are not available in the public health sector of SA.^[1,7]^

Ms PM’s response to therapy was monitored via serial chest X-ray imaging, which showed progressive resolution of cavitatory lesions (Fig. 3). Serial radiographic changes and sCLAT titres reliably reflect disease progression and the response to therapy.^[2,5]^ Ms PM continued with the therapeutic dose of fluconazole until resolution of lung infiltrates (total of 6 months). HIV-infected patients should continue chronic maintenance therapy with fluconazole (200 mg per day), until the CD4 count is greater than 200 cells/ μL, with a suppressed viral load, and a sCLAT titre persistently ≤1:512.^[1,7]^ Surgical excision of infected pulmonary tissue is only indicated in cases of masses that impinge on adjacent structures, or in infected patients with poor response to maximal medical therapy.^[1]^

## Conclusion

The incidence of pulmonary cryptococcosis in the context of the HIV pandemic in SA is unknown. Although antiretroviral therapy (ART) is now accessible in SA, many HIV-infected patients not on ART with CD4 counts <100 cells/μL may present to hospital for admission with primarily respiratory symptoms. Therefore, physicians should have a high index of suspicion for pulmonary cryptococcosis with dissemination. The observation of multiple lung nodules or cavities not explained by any other disease on chest imaging should raise the suspicion for pulmonary cryptococcosis even further. Sputum collection for culture, and where possible bronchoscopic lavage and tissue specimens, should be sent to the laboratory for evaluation, to further corroborate the diagnosis.

In our setting, fluconazole therapy is accessible and should be initiated immediately once the diagnosis is made. Intravenous amphotericin B is indicated if co-existing CCM is also diagnosed. Duration of therapy should be individualised, with response to therapy monitored by serial chest images via chest X-ray and contrast CT scan of the chest, and where possible sCLAT titre measurements. Pulmonary cryptococcosis is a treatable disease with good outcomes if treated timeously. The addition of ART in our HIV-infected patient was paramount in preventing opportunistic infections such as pulmonary cryptococcus, but physicians must be alert in recognising and adequately managing the development of unmasking or paradoxical IRIS, in the case of disseminated cryptococcal disease.
